# *FOCUS*: fast Monte Carlo approach to coherence of undulator sources

**DOI:** 10.1107/S1600577522010748

**Published:** 2023-01-01

**Authors:** M. Siano, G. Geloni, B. Paroli, D. Butti, T. Lefèvre, S. Mazzoni, G. Trad, U. Iriso, A. A. Nosych, L. Torino, M. A. C. Potenza

**Affiliations:** a Dipartimento di Fisica, Università degli Studi di Milano, INFN Sezione di Milano, and CIMAINA, via G. Celoria 16, 20133 Milan, Italy; b European XFEL, Holzkoppel 4, 22869 Schenefeld, Germany; c CERN, CH-1211 Geneva, Switzerland; d ALBA-CELLS Synchrotron Radiation Facility, Carrer de la Llum 2-26, 08290 Cerdanyola del Valles, Barcelona, Spain; University of Tokyo, Japan

**Keywords:** undulator sources, cross spectral density function, spectral degree of coherence, degree of coherence, GPU, synchrotron radiation, coherence, partially coherent radiation, Monte Carlo simulations, numerical algorithms

## Abstract

*FOCUS* (*Fast Monte CarlO approach to Coherence of Undulator Sources*) is a new GPU-based simulation code to compute the transverse coherence of undulator radiation from ultra-relativistic electrons. The core structure of the code, which is written in the language C++ accelerated with CUDA, combines an analytical description of the emitted electric fields and massively parallel computations on GPUs. The combination is rigorously justified by a statistical description of synchrotron radiation based on a Fourier optics approach. *FOCUS* is validated by direct comparison with multi-electron *Synchrotron Radiation Workshop* (*SRW*) simulations, evidencing a reduction in computation times by up to five orders of magnitude on a consumer laptop. *FOCUS* is then applied to systematically study the transverse coherence in typical third- and fourth-generation facilities, highlighting peculiar features of undulator sources close to the diffraction limit. *FOCUS* is aimed at fast evaluation of the transverse coherence of undulator radiation as a function of the electron beam parameters, to support and help prepare more advanced and detailed numerical simulations with traditional codes like *SRW*.

## Abstract 2

*FOCUS* (*Fast Monte CarlO approach to Coherence of Undulator Sources*), a new GPU-based code to compute the transverse coherence of X-ray radiation from undulator sources as a function of the electron beam parameters, is described. *FOCUS* is validated with the *Synchrotron Radiation Workshop* (*SRW*) and *SPECTRA* codes. Examples of application to coherence studies in third- and fourth-generation light sources are shown.

## 1. Introduction

The smaller and smaller electron beam emittances recently achieved in third- and fourth-generation synchrotron light sources have naturally drawn attention to the necessity of describing the generation and propagation of partially coherent light pulses (Liu & Westfahl, 2017 ▸; Shin, 2021 ▸).

In fact, electron beams with emittances much smaller than the radiation wavelength generate diffraction-limited light that can be effectively described in terms of wave optics. The opposite limit of fully transversely incoherent light is realized when the beam emittance is much larger than the radiation wavelength, and is satisfactorily modeled by geometrical optics. Situations between these two limits are referred to as partially coherent. In this case, a statistical description based on wave optics is required since the pulse wavefront undergoes random changes from pulse to pulse, ultimately due to the shot-noise in the electron beam (Born & Wolf, 1970 ▸; Mandel & Wolf, 1995 ▸; Goodman, 2000 ▸).

Within the realm of statistical optics, partially coherent light is naturally described by field correlation functions (Born & Wolf, 1970 ▸; Mandel & Wolf, 1995 ▸; Goodman, 2000 ▸). In the space–frequency domain, and assuming that the radiation wavelength is much shorter than the electron bunch length (longitudinally incoherent emission), this amounts to the specification of the so-called cross-spectral density (CSD) correlating the slowly varying amplitude of the Fourier transform of the electric field (in short, the field) at two different spatial positions (Born & Wolf, 1970 ▸; Mandel & Wolf, 1995 ▸; Goodman, 2000 ▸).

Similarly to the electric field, the CSD can, in principle, be propagated through an X-ray beamline, to characterize the transverse coherence properties of the emitted light at any longitudinal position from the radiation source (Born & Wolf, 1970 ▸; Mandel & Wolf, 1995 ▸; Goodman, 2000 ▸). This has become of utmost importance in modern synchrotron facilities exploiting coherence-based techniques such as X-ray photon correlation spectroscopy, coherent diffraction imaging, propagation-based phase-contrast imaging and ptychography (Nugent, 2010 ▸). For example, transverse coherence directly impacts the ability to efficiently focus the X-ray beam to the nanometre range (Singer & Vartanyants, 2014 ▸), as well as to reach sub-nanometre resolutions in X-ray imaging techniques (Schroer & Falkenberg, 2014 ▸).

In order to describe modern X-ray beamlines, different approaches with increasing level of complexity can be adopted, from simple analytical estimations to detailed numerical simulations based either on ray-tracing or on wave optics (Sanchez del Rio *et al.*, 2019 ▸). To this aim, there exist a number of well known and established codes such as *Synchrotron Radiation Workshop* (*SRW*) (Chubar & Elleaume, 1998 ▸; Chubar, 2014 ▸), *SPECTRA* (Tanaka & Kitamura, 2001 ▸; Tanaka, 2021 ▸), *X-Ray Tracer* (*XRT*) (Klementiev & Chernikov, 2014 ▸), *SHADOW3* (del Rio *et al.*, 2011 ▸) and *COherent Modes for SYnchrotron Light (COMSYL* ) (Glass & Sanchez del Rio, 2017 ▸), to name a few. Perhaps the most advanced and widespread wave optics code is *SRW*, a high-accuracy general computer code for synchrotron radiation sources. It has been extensively benchmarked in different synchrotron radiation facilities and has become widely accepted within the accelerator and the X-ray optics communities. Codes like *SRW* are based upon the paraxial approximation, motivated by the fact that one deals with ultra-relativistic electrons, but they are otherwise very general.

Notwithstanding the availability of accurate wave optics codes, calculation and propagation of the CSD is very cumbersome, from a computational point of view. The partial coherence of the emitted radiation demands multi-electron simulations for accurate analysis. This is required even for the upcoming ultralow-emittance storage rings, since the emitted radiation cannot be fully described by a single, perfectly coherent wavefront in a large spectral range (Walker, 2019 ▸; Khubbutdinov *et al.*, 2019 ▸). In such cases, parallelization is usually adopted to speed up wavefront propagation calculations. For example, *SRW* allows parallelization on the CPU based on the message passing interface (MPI) or on the open multi-processing (OpenMP). The *SRW* MPI parallelization exhibits a good scaling with the number of parallel processes but tends to over-consume memory, whereas the OpenMP parallelization is more memory-efficient but requires multi-core servers for proper scaling (He *et al.*, 2020 ▸). Alternatively, several libraries offer GPU-oriented optimized algorithms to perform operations like fast Fourier transforms (FFTs) and matrix multiplications, which are at the basis of numerical wavefront propagation through arbitrary optical elements. This enables relatively fast wavefront propagation computations by exploiting the large number of processing cores of modern graphics cards. However, depending on the complexity of the beamline, multi-electron simulations are usually long-running and computationally expensive, and it is not possible to know *a priori* how many electrons must be sampled to obtain a given level of accuracy.

One of the most effective solutions to these problems used in modern codes is based on the expansion of the original CSD in coherent modes, which goes under the name of coherent mode decomposition (Mandel & Wolf, 1995 ▸; Glass & Sanchez del Rio, 2017 ▸; Singer *et al.*, 2008 ▸; Vartanyants & Singer, 2010 ▸). Once the modes themselves are found, they can be propagated separately, and their subsequent random addition generates a field realization. This approach is particularly advantageous for highly coherent radiation described by a few modes only, whereas it becomes computationally inefficient as the number of required modes increases. Furthermore, coherent mode decomposition is costly both in terms of computer resources (GB to TB of memory) and computation times (many hours on multi-core computer clusters), though recent progress based on an analytic treatment of common quadratic phase factors (Chubar & Celestre, 2019 ▸; Li & Chubar, 2022 ▸) and factorization of the CSD (Sanchez del Rio *et al.*, 2022 ▸) are promising.

The coherent mode representation of the CSD, being very general, provides an excellent theoretical insight into the problem. Nonetheless, the coherent modes are not easy to determine in practice (Flewett *et al.*, 2009 ▸), and most experiments directly assess the profiles (Leitenberger *et al.*, 2004 ▸; Vartanyants *et al.*, 2011 ▸; Singer *et al.*, 2012 ▸; Skopintsev *et al.*, 2014 ▸; Pfeiffer *et al.*, 2005 ▸; Snigireva *et al.*, 2001 ▸; Lyubomirskiy *et al.*, 2016 ▸) or the 2D map (Alaimo *et al.*, 2009 ▸, 2014 ▸; Kashyap *et al.*, 2015 ▸; Siano *et al.*, 2015 ▸, 2017 ▸, 2021 ▸, 2022 ▸) of the normalized CSD, known in statistical optics as the spectral degree of coherence (SDC) (Born & Wolf, 1970 ▸; Mandel & Wolf, 1995 ▸; Goodman, 2000 ▸).

In this paper we describe a simulation code natively running on GPUs for fast and accurate evaluation of the SDC of undulator radiation propagating in free space. The code is named *FOCUS* (*Fast Monte CarlO approach to Coherence of Undulator Sources*). We advantageously combine an analytical description of the emitted electric fields and a rigorous statistical treatment of synchrotron radiation to expose parallelism and harness the compute capabilities of modern GPUs. The analytical expressions for the emitted electric fields are derived under specific assumptions within a Fourier optics treatment of synchrotron radiation from ultra-relativistic electrons (Geloni *et al.*, 2007 ▸). This unavoidably poses some limitations to the applicability range of the code, which trades generality for calculation speed. Within its applicability domain, *FOCUS* achieves a considerable reduction in computation times by several orders of magnitude with respect to standard multi-electron *SRW* simulations. This enables fast and thorough simulations with millions of particles in a few seconds on a consumer laptop. Unlike other codes capable of simulating entire beamlines, *FOCUS* is dedicated to the accurate characterization and modeling of the transverse coherence properties of synchrotron radiation from undulator sources as a function of the electron beam parameters. Besides being certainly of interest on a general theoretical basis (Walker, 2019 ▸; Khubbutdinov *et al.*, 2019 ▸; Geloni *et al.*, 2008 ▸), this is also of practical relevance, *e.g.* for electron beam diagnostics based on interferometric techniques (Siano *et al.*, 2021 ▸, 2022 ▸), or to assess the impact of partial coherence on the performances of focusing X-ray optics (Singer & Vartanyants, 2014 ▸; Schroer & Falkenberg, 2014 ▸; Sanchez del Rio *et al.*, 2019 ▸; Thomas *et al.*, 2016 ▸). In addition, it is worth mentioning that the proposed approach can be adapted to imaging geometries as well, by studying conjugate planes with the proper magnification factor (Alaimo *et al.*, 2009 ▸; Thomas *et al.*, 2016 ▸; Goodman, 2007 ▸). The code then is complementary to the existing ones. It is aimed at fast evaluating the transverse coherence properties of undulator radiation, in such a way that the main source parameters and their range of variability can be easily identified. In this view, *FOCUS* can support and help prepare more advanced and detailed numerical simulations with traditional codes like *SRW*, and time-consuming simulations can be run only when truly necessary.

The paper is organized as follows. In Section 2 we review the theory underlying *FOCUS*. In Section 3 we describe the general structure of the code, while in Section 4 we benchmark *FOCUS* with *SRW*. In Sections 5 and 6 we apply *FOCUS* to the accurate characterization of the transverse coherence properties of typical third- and fourth-generation facilities, respectively, highlighting peculiar features especially for undulator sources close to the diffraction limit. Finally, we collect our conclusions in Section 7.

## 2. Theory

### 2.1. Fourier optics approach to undulator radiation

Let 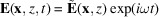 be the analytic representation in the space–time domain of a monochromatic electric field emitted by an electron moving through a planar undulator. The radiation has angular frequency ω and wavelength λ = 2π*c*/ω. Here **x** = (*x*, *y*) denotes transverse positions across the observation plane, *z* is the distance from the undulator center, and *t* is the time. The complex amplitude 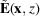 describes the electric field in the space–frequency domain (rigorously, apart from a Dirac delta function to ensure correct physical dimensions). It can always be written as 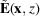 = 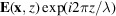 , where **E**(**x**, *z*) is the envelope of the field in the space–frequency domain. In the following, we will refer to **E**(**x**, *z*) simply as the field for notation simplicity.

We will derive analytical expressions for **E**(**x**, *z*) valid for ultra-relativistic electrons moving through an ideal undulator and under the so-called resonant approximation 4π*N*_*w*_ ≫ 1, *N*_*w*_ being the number of undulator periods. We notice that, in principle, our approach can be adapted to account for magnetic field errors as well, for example by computing the Fourier spectrum of the magnetic field inside the undulator device and by generalizing our equations to each Fourier component of the magnetic field (possibly by also relaxing the resonant approximation). However, in practice, this might be cumbersome from a numerical implementation viewpoint and result in a highly inefficient code. Finally, we would like to remark that this is outside the scope of *FOCUS*, whose specific aim is to provide an easy simulation tool, complementary to existing ones, for fast computation of the coherence properties of undulator radiation as a function of the electron beam parameters. Therefore, in the following, we will restrict to the case of an undulator source in the absence of magnetic field errors.

In the ultra-relativistic regime γ ≫ 1, γ being the Lorentz factor, **E**(**x**, *z*) varies slowly with respect to the wavelength. Thus, **E**(**x**, *z*) can be obtained by solving paraxial Maxwell equations in the space–frequency domain by means of a parabolic Green’s function (Weinberger, 1965 ▸). Under the resonant approximation, we can neglect terms proportional to the gradient of the charge density in the solution to the paraxial wave equation, as well as the entire vertical polarization component (Geloni *et al.*, 2007 ▸). Therefore, for frequencies near the undulator harmonics, the field at the observation plane is a complex scalar quantity described by the general expression (Geloni *et al.*, 2007 ▸, 2018 ▸) 

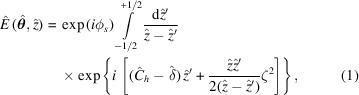

where we have defined reduced quantities and dimensionless parameters as 

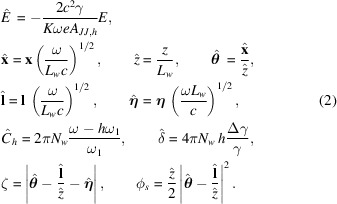

In equations (2), *c* is the speed of light, *K* is the undulator strength parameter, −*e* is the electron charge, *h* is the harmonic number of the radiation emitted from the undulator, *A*_*JJ*,*h*_ = (−1)^(*h*−1)/2^[*J*_(*h*−1)/2_(*u*) − *J*_(*h*+1)/2_(*u*)], *u* = *hK* ^2^/[2(2 + *K* ^2^)], *J*_*n*_ is the Bessel function of the first kind of order *n*, *L*_*w*_ = *N*_*w*_λ_*w*_ is the undulator length, λ_*w*_ is undulator period, ω_1_ = 4πγ^2^ *c*/[λ_*w*_(1 + *K* ^2^/2)] is the first harmonic of the undulator, **l** and **η** are the electron offset and deflection, respectively, and Δγ/γ is the relative energy deviation due to the finite energy spread of the electron beam.

Introduction of dimensionless quantities according to equations (2) reduces the parameters involved in the description of real systems to a few universal quantities with physical relevance. This enables scalability among different cases of practical interest. In particular, equations (2) amount to normalize spatial and angular quantities to the diffraction size (*L*_*w*_ *c*/ω)^1/2^ and diffraction angle (*c*/ω*L*_*w*_)^1/2^ of single-electron radiation, distances to the undulator length, and detuning and energy spread to the undulator resonant bandwidth.

Equation (1) is a general expression in paraxial approximation valid in the far-field as well as in the near-field. For 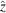 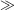 1 (Fraunhofer zone or far-field zone), we neglect terms of order higher than 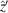 in the expansion of 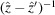 in the phase of equation (1), which can therefore be integrated analytically leading to (Geloni *et al.*, 2007 ▸) 

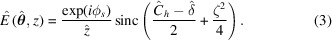

The field in the Fraunhofer zone is a diverging spherical wave originating from the center of the undulator and modulated in amplitude by the sinc(α) term describing the resonant character of the undulator device. Notice that the particle offset 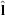 and deflection 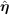 are effective in shifting and tilting the far-field pattern.

Thanks to the ultra-relativistic regime, paraxial approximation can always be applied and we can describe the far-field radiation given in equation (3) in terms of Fourier optics (Goodman, 2007 ▸). In particular, undulator radiation from an ultra-relativistic electron is interpreted as a laser-like beam originating from a virtual source located at the center of the undulator and exhibiting a plane wavefront, similarly to the waist of a laser beam (Geloni *et al.*, 2007 ▸). The electric field distribution of such a virtual source is related to the inverse Fourier transform of the far-field pattern in equation (3), and has a characteristic transverse extent (*L*_*w*_ *c*/ω)^1/2^ corresponding to a natural diffraction angle (*c*/*L*_*w*_ω)^1/2^ (Geloni *et al.*, 2007 ▸). This ultimately lays the foundations of the transverse and angular scalings in equations (2).

The field at the virtual source is then propagated at any 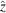 by means of the paraxial Fresnel propagation formula. At perfect resonance 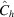 = 0 and in the absence of energy spread this leads to (Geloni *et al.*, 2007 ▸) 

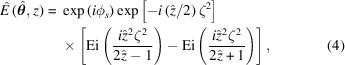

where Ei is the exponential integral function (Abramowitz & Stegun, 1964 ▸). Equation (4) is valid at any longitudinal position 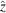 downstream of the undulator, and reduces to equation (3) for 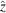 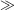 1.

### 2.2. Statistical description of synchrotron radiation

The field generated by an electron beam with finite emittance composed by *N*_e_ electrons is given by 

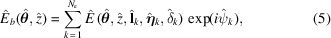

where 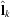 , 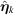 and 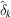 are the offset, deflection and relative energy deviation of the *k*th electron, and 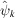 = 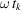 is the phase retardation associated with the random arrival time *t*_*k*_ of the *k*th electron.

The transverse coherence properties of undulator radiation are described in the space–frequency domain by the so-called CSD (Born & Wolf, 1970 ▸; Mandel & Wolf, 1995 ▸; Goodman, 2000 ▸),

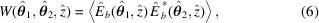

where angular brackets denote the ensemble average.

The CSD is related to the spectral density (SD) by (Born & Wolf, 1970 ▸; Mandel & Wolf, 1995 ▸; Goodman, 2000 ▸) 

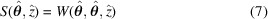

and to the SDC by (Born & Wolf, 1970 ▸; Mandel & Wolf, 1995 ▸; Goodman, 2000 ▸) 

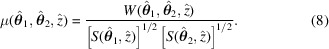

Substitution of equation (5) into equation (6) leads to 

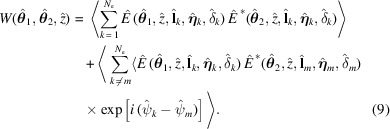

In the case of an electron beam much longer than the radiation wavelength, the electron arrival times are not correlated with each other, hence the second summation vanishes upon the ensemble average. As a result, the CSD, SD and SDC are expressed as a function of single-electron terms only (Geloni *et al.*, 2008 ▸), 

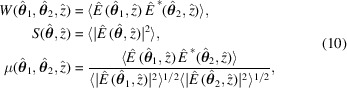

where the ensemble averages are performed over the phase space density 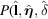 ) = 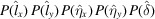 of the electron beam at the undulator center. We assume Gaussian probability density functions with variances given in normalized units by (Geloni *et al.*, 2008 ▸) 

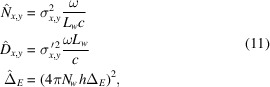

where σ_*x*,*y*_ and 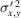 are the horizontal and vertical r.m.s. electron beam size and divergence, respectively, and Δ_*E*_ is the r.m.s. energy spread of the electron beam. Definition of the reduced parameters in equation (11) follows directly from equations (2).

Finally, a useful figure of merit to quantify the coherence properties of synchrotron radiation by a single number is the degree of coherence (Born & Wolf, 1970 ▸; Mandel & Wolf, 1995 ▸; Goodman, 2000 ▸),

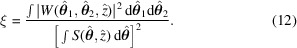

Values ξ = 1 and ξ = 0 correspond to fully coherent and fully incoherent radiation, respectively.

## 3. Structure of the code

*FOCUS* calculates the ensemble averages in equations (10), namely 5D integrals over the electron beam phase space density, by means of a Monte Carlo approach (Press *et al.*, 1992 ▸),

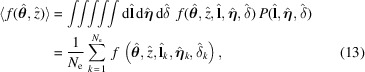

where 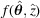 denotes quantities between brackets on the right-hand side of equations (10) and *N*_e_ is the number of electrons in the beam.

As we have previously mentioned, each term in the summations is due to the *k*th electron only, and it does not depend on the other contributions. This makes the adopted approach particularly suitable for massively parallel implementations, and allows harnessing the computation capabilities of modern GPUs to perform fast and thorough simulations. In addition, the calculation speed is further increased by adopting the analytical framework based on equations (3) and (4). The unique combination of these two approaches stands at the core of *FOCUS*.

**Figure 1:**
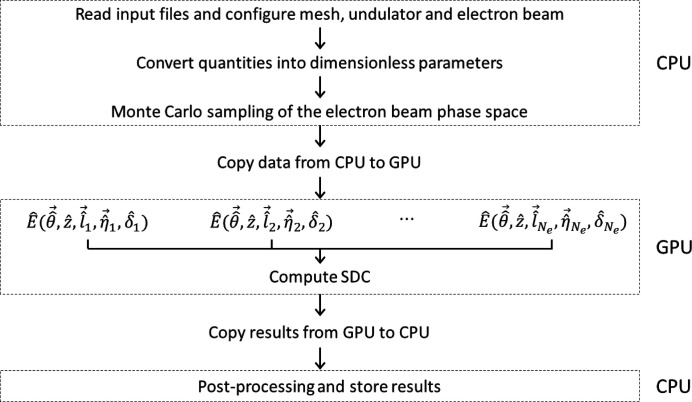
General workflow of *FOCUS*.

*FOCUS* is written in C++ language accelerated with CUDA to harness the compute capabilities of modern NVIDIA graphics cards, and performs double-precision calculations for high-accuracy results. The general workflow is sketched in Fig. 1 ▸, where we highlight operations running either on the CPU or on the GPU.

First, the main parameters required to run the simulation (mesh size and resolution, longitudinal position of the observation plane, undulator and electron beam parameters) are imported from external files, which makes data input quite easy and flexible. Parameters are then converted into dimensionless quantities according to equations (2). The 5D phase space density of the electron beam is sampled either by an internal method relying on random number generators (Press *et al.*, 1992 ▸) or by reading values from a user-supplied file. This latter option is useful for benchmark purposes, as well as to assess the influence of non-Gaussian beams on the transverse coherence properties.

Computation is then moved from the CPU onto the GPU by copying data on the graphics card memory with synchronous memory transfers. The GPU computes each term of the summations in equation (13) in parallel, through the concurrent execution of many threads on the CUDA processors residing on the graphics card. To optimize the GPU resources, the number of threads and their organization are determined at a runtime based on *N*_e_. This also ensures maximization of the overall throughput of the graphics card, since a new thread is executed as soon as one CUDA processor ends its task. The final sum in equation (13) is also implemented in a parallel algorithm running on the GPU. A synchronization barrier is required to prevent the GPU from accessing memory blocks that are still being processed. Notice that the massively parallel computations are performed on data that reside on the graphics card memory to avoid communication bottlenecks caused by the continuous (slow) data transfers between CPU and GPU.

Finally, results are copied back to the CPU for final post-processing and storage. Stored results are formatted as text files complying with most data visualization tools.

## 4. Benchmark with *SRW*

As a first benchmark, we run *FOCUS* and *SRW* for a single electron and for an electron beam with zero emittance and zero energy spread (a filament beam), which, by definition, emit radiation endowed with full coherence. In addition, this benchmark is essential to prove that the massively parallel computations of *FOCUS* running on the GPU are correctly processing data, since the CSD must equal *N*_e_ times the single-electron intensity distribution for 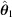 = 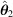 . The wavefronts simulated with both *FOCUS* and *SRW* are fully coherent with 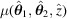 = 1 regardless of 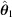 , 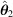 and 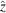 . Random numerical fluctuations in the order of 10^−7^–10^−8^ are present in the *SRW* results due to the rounding of single-precision numbers. In contrast, *FOCUS* simulations have higher accuracy thanks to the double-precision floating-point operations.

We then perform a direct comparison between *FOCUS* and *SRW* for the case of an electron beam with finite emittance and energy spread. To this aim, we consider the NCD-SWEET undulator source at the ALBA Synchrotron Light Source as our case study representative of third-generation synchrotron light sources (Siano *et al.*, 2022 ▸). The main parameters of the NCD-SWEET beamline are summarized in Table 1 ▸. Other facilities can be described as well, by properly scaling and tailoring physical quantities according to equations (2).

**Figure 2:**
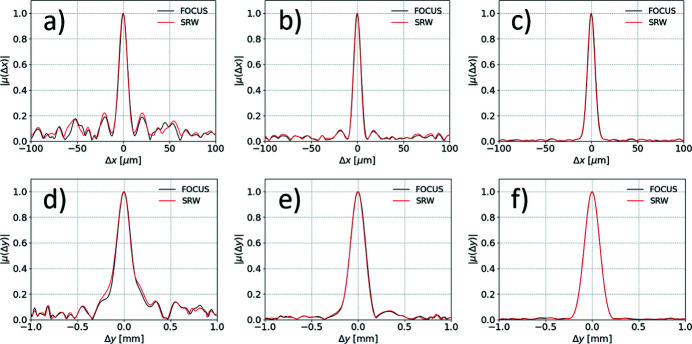
Direct comparison between *FOCUS* and *SRW* simulations for the horizontal (*a*,*b*,*c*) and vertical (*d*,*e*,*f*) profiles of the SDC of a typical third-generation undulator source. Results with *N*_e_ = 10^3^ (*a*,*d*), *N*_e_ = 10^4^ (*b*,*e*) and *N*_e_ = 10^5^ (*c*,*f*) are shown.

We perform simulations with *N*_e_ varying from 10 to 10^5^. The same electron offsets, deflections and relative energy deviations are used in both *FOCUS* and *SRW* for a one-to-one comparison. Examples of the simulated horizontal and vertical profiles of the SDC are reported in Fig. 2 ▸. *FOCUS* results match *SRW* simulations, accurately reproducing even the spurious fluctuations of the SDC at large Δ*x* and Δ*y* due to the relatively low number of simulated electrons. Results also show that a large number of electrons of the order of 10^5^ or larger is required to suppress such spurious contributions.

We report a comparison of simulation times in Table 2 ▸. *FOCUS* performances refer to a consumer laptop mounting a NVIDIA GeForce 940MX graphics card with 512 CUDA cores (2 GB dedicated graphics memory), while *SRW* simulations are run with multi-threading parallelization on the dual-core CPU (Intel Core i7, 2.5 GHz, 8 GB DDR4 RAM). The comparison shows that *FOCUS* is faster by four to five orders of magnitude. Furthermore, the execution time of *FOCUS* is a non-linear function of the number of electrons in the beam, opposite to the *SRW* case. Thanks to the parallel computation of the different terms in equation (13), it is practically constant for *N*_e_ ≤ 10^4^, and scales linearly with the number of electrons, as in the *SRW* case, only when all GPU resources are fully utilized, as for example when *N*_e_ is increased to 10^6^ or larger. In this case, the *FOCUS* computation time is 3–4 s, while *SRW* simulations would take 4–5 days. More modern, high-end GPUs with more CUDA cores and dedicated memory can in principle achieve even better performances.

*FOCUS* performances are due to the peculiar adopted approach combining massively parallel computations on GPUs with an analytical description of the electric fields. In particular, for the specific GPU with 512 CUDA cores used during these tests, the overall speedup by five orders of magnitude equally results from the GPU-based computations and the analytical framework.

Finally, one last comment is worth making concerning the applicability of the code to the case of undulator radiation off resonance, when the intensity distribution exhibits a ring shape. In principle, *FOCUS* allows computation of the transverse coherence properties of detuned undulator radiation thanks to the detuning term 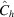 in the expression of equation (3) for the electric field generated by individual electrons. In practice, however, one must recall the conditions under which such an expression was derived, namely the resonant approximation (requiring a large number of undulator periods *N*_*w*_), which limits the rigorous applicability of the adopted analytical framework to frequencies near resonance. In particular, 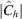 should be compared, parametrically, with *N*_*w*_ and be much smaller than it. For 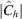 ≃ 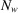 or larger, one might observe deviations from more accurate simulation tools like *SRW*, depending on the actual values of the parameters. For the NCD-SWEET undulator source considered here, discrepancies are limited to below the 10% level for a relative detuning of roughly 1% (comparable with the natural bandwidth of the undulator radiation) from the *h* = 7 harmonic, corresponding to 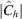 = 40. Such deviations from *SRW* results progressively decrease, and eventually vanish, for smaller and smaller 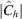 .

## 5. Systematic study of transverse coherence of a typical third-generation synchrotron light source

In third-generation facilities, undulator sources are usually described as quasi-homogeneous fully incoherent thermal sources, and estimations of the transverse coherence properties rely on the application of the well known van Cittert and Zernike theorem, which relates the SDC to the Fourier transform of the source intensity distribution (Born & Wolf, 1970 ▸; Mandel & Wolf, 1995 ▸; Goodman, 2000 ▸). However, the applicability of the van Cittert and Zernike theorem is highly debated, especially along the vertical direction when the electron beam emittance ε_*y*_ = 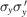 becomes comparable with the natural photon beam emittance ε_ph_ = λ/2π (Geloni *et al.*, 2008 ▸; Alaimo *et al.*, 2009 ▸; Thomas *et al.*, 2016 ▸). The topic is of high theoretical and practical interest, as shown by a number of recent publications (Walker, 2019 ▸; Khubbutdinov *et al.*, 2019 ▸; Geloni *et al.*, 2008 ▸; Alaimo *et al.*, 2009 ▸; Siano *et al.*, 2022 ▸; Thomas *et al.*, 2016 ▸).

Here we systematically study the transverse coherence properties of a typical third-generation synchrotron light source by considering the same undulator setup as in the previous section. We perform accurate *FOCUS* simulations with *N*_e_ = 10^6^–10^7^ to limit relative fluctuations in the results to 0.1% or less.

**Figure 3:**
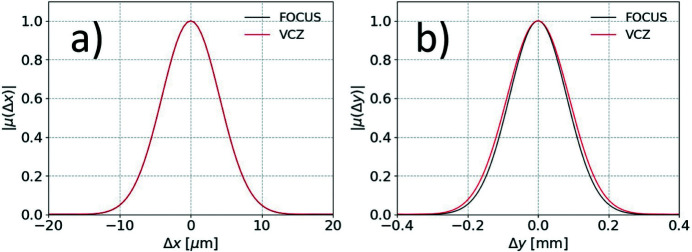
Horizontal (*a*) and vertical (*b*) profiles of the SDC for a typical third-generation synchrotron light source. Predictions based on the van Cittert and Zernike theorem (VCZ) are also reported for direct comparison.

**Figure 4:**
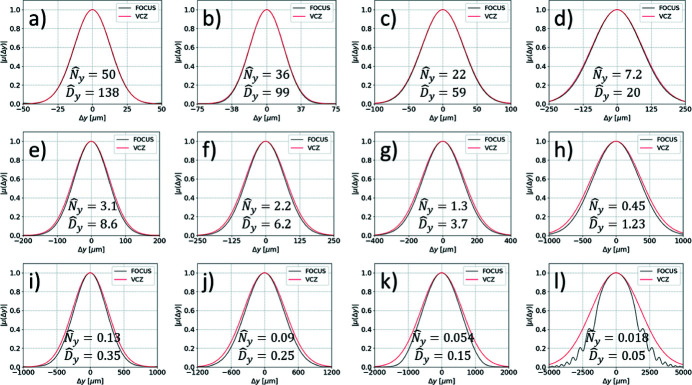
Systematic study of the vertical coherence for a typical third-generation synchrotron light source for radiation tuned at the harmonic *h* = 7 with λ = 0.1 nm (*a*,*e*,*i*), *h* = 5 with λ = 0.14 nm (*b*,*f*,*j*), *h* = 3 with λ = 0.23 nm (*c*,*g*,*k*), and *h* = 1 with λ = 0.7 nm (*d*,*h*,*l*), and for decreasing vertical beam size σ_*y*_ = 40 µm (*a*,*b*,*c*,*d*), σ_*y*_ = 10 µm (*e*,*f*,*g*,*h*), and σ_*y*_ = 2 µm (*i*,*j*,*k*,*l*). Predictions based on the van Cittert and Zernike theorem (VCZ) are also reported for direct comparison. The electron beam divergence is varied according to 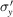 = σ_*y*_/β_*y*_ where β_*y*_ = 1.2 m is the vertical beta function at the undulator center. In each plot we also report the corresponding normalized parameters 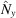 and 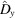 according to equation (11).

We report in Fig. 3 ▸ results for the simulated horizontal and vertical profiles of the SDC for the undulator source with nominal parameters as in Table 1 ▸, while in Fig. 4 ▸ we show a systematic investigation of the vertical coherence obtained by varying the radiation wavelength and the vertical beam emittance. Predictions based on the van Cittert and Zernike theorem are also reported alongside for direct comparison.

Results show that the quasi-homogeneous assumption is valid along the horizontal direction, where simulated data and the analytical model based on the van Cittert and Zernike theorem perfectly agree. The same is valid along the vertical direction for relatively large beam sizes of the order of tens of micrometres, corresponding to 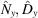 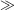 1 in dimensionless units. Contrarily, the van Cittert and Zernike theorem does not accurately describe the transverse coherence of the undulator source along the vertical direction for beam sizes as small as a few micrometres, and discrepancies with the more rigorous statistical optics approach arise. This stems from the fact that the electron beam emittance becomes comparable with, or smaller than, the photon beam emittance. In dimensionless units, this corresponds to 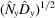 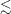 1. Therefore, undulator sources along the vertical direction cannot in general be regarded as quasi-homogeneous fully incoherent thermal sources.

As shown in Fig. 4 ▸, deviations are more evident as 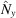 and 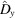 decrease. Notice that the vertical coherence also exhibits a markedly non-Gaussian behavior for extremely small 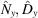 , which is related to the oscillatory behavior of the sinc(α) function in the single-electron radiation field, as we will detail in the next section. Therefore, while the observed discrepancies are small in current third-generation machines, they are however indicative of deviations which will become more evident in future fourth-generation light sources, especially close to the diffraction limit. In such situations, numerical codes like *FOCUS* represent an indispensable tool for proper radiation diagnostics.

## 6. Applications to fourth-generation synchrotron light sources

To showcase the peculiar coherence properties of fourth-generation synchrotron light sources close to the diffraction limit, we consider radiation with λ = 2.5 nm emitted by an undulator source with 10 pm emittance in both horizontal and vertical directions. The main parameters are summarized in Table 3 ▸, and are compatible with the PETRA IV case (Schroer *et al.*, 2018 ▸). We notice that the same radiation source has been the object of similar numerical investigations in a recent publication (Khubbutdinov *et al.*, 2019 ▸), which we refer to as an additional benchmark of the *FOCUS* code in case of ultralow emittances.

**Figure 5:**
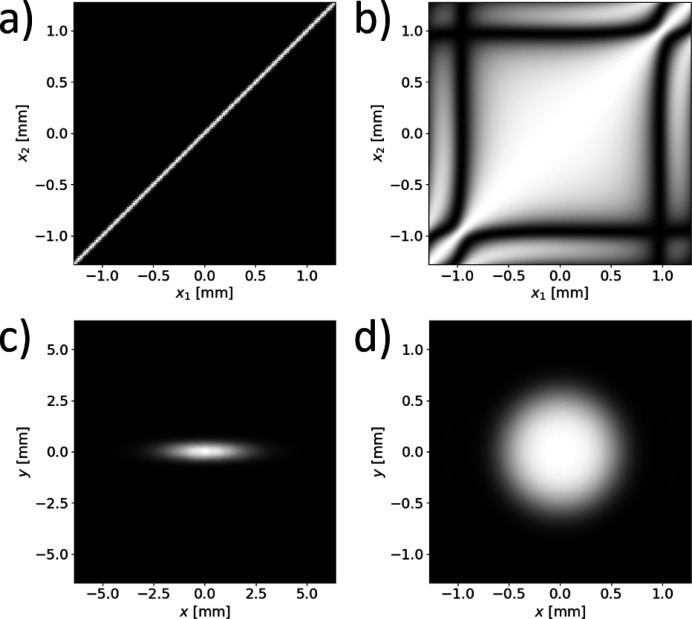
2D map of the horizontal SDC as a function of *x*_1_ and *x*_2_ for a typical third-generation synchrotron light source (*a*) and for a fourth-generation synchrotron light source close to the diffraction limit (*b*). The vertical position of the two observation points is *y*_1_ = *y*_2_ = 0. The corresponding SDs are reported in (*c*) and (*d*), respectively.

**Figure 6:**
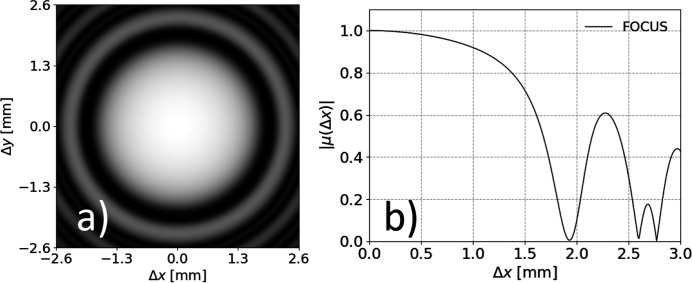
2D map of the on-axis SDC as a function of Δ*x* and Δ*y* (*a*), and corresponding horizontal profile at Δ*y* = 0 (*b*), for a typical fourth-generation synchrotron light source close to the diffraction limit.

We report in Fig. 5 ▸ a detailed analysis of the horizontal coherence properties as a function of *x*_1_ and *x*_2_. The vertical position of the two observation points is fixed on-axis at *y*_1_ = *y*_2_ = 0. For comparison, we also show the results of similar simulations for the third-generation undulator source described in the previous sections. In both cases, we report results for the SDC and the SD, from which the degree of coherence is inferred based on equations (12). In Fig. 6 ▸ we characterize the full 2D coherence properties on-axis, at (**x**_1_ + **x**_2_)/2 = (0, 0), as a function of Δ**x** = **x**_1_ − **x**_2_ = (Δ*x*, Δ*y*).

For the ALBA case, the horizontal and vertical degree of coherence are ξ_*x*_ = 1.250 × 10^−3^ and ξ_*y*_ = 0.118, respectively. Results are in good agreement with independent *SPECTRA* simulations yielding ξ_*x*_ = 1.252 × 10^−3^ and ξ_*y*_ = 0.115. For the PETRA IV case, ξ_*x*_ = ξ_*y*_ = 0.917 in the absence of energy spread. Results are compatible with values reported in the literature (Khubbutdinov *et al.*, 2019 ▸). By adding a finite energy spread of 10^−3^, we find a reduction of the degree of coherence to ξ_*x*_ = ξ_*y*_ = 0.884, as also reported in recent publications (Geloni *et al.*, 2018 ▸; Khubbutdinov *et al.*, 2019 ▸).

As shown by Fig. 5 ▸, the coherence properties of a fourth-generation synchrotron light source close to the diffraction limit are highly dependent on the absolute position across the observation plane. This is reflected in Fig. 5 ▸ by the squared shape of the horizontal SDC. Contrarily, in third-generation synchrotron light sources far from the diffraction limit, coherence properties depend only on the relative distance between the two observation points (Δ*x* = *x*_1_ − *x*_2_ in this case).

We also notice the presence of deep oscillations in the 2D coherence maps in Figs. 5 ▸ and 6 ▸. They can be ascribed to the oscillatory behavior of the sinc(α) modulation of the single-particle electric field, which affects the transverse coherence in the presence of a finite, albeit small, emittance, according to a mechanism recently described (Geloni *et al.*, 2018 ▸). In fact, due to the finite emittance, different electrons generate different wavefronts at the observation plane. In particular, the field from different electrons changes sign at different positions, according to equations (3) and (4). This results in an effective change in the wavefronts, which by definition impacts on the coherence properties (Geloni *et al.*, 2018 ▸). In particular, the sign reversals induce anti-correlations in the complex fields, which in turn results in deep modulations of the SDC. The oscillations in the 2D coherence maps in Figs. 5 ▸ and 6 ▸ are therefore a clear indication that the coherence properties of undulator sources close to the diffraction limit are simultaneously affected by the beam emittance and the peculiar features of the single-electron radiation.

## 7. Conclusions

We have described *FOCUS* (*Fast Monte CarlO approach to Coherence of Undulator Sources*), a new simulation code natively running on NVIDIA GPUs to compute the transverse coherence of synchrotron radiation from ultra-relativistic electrons in an undulator source.

The code relies on analytic expressions for the emitted electric fields derived with a Fourier optics formulation of synchrotron radiation, combined with massively parallel computations on GPUs. A consistent use of suitable dimensionless parameters reduces the variables involved in the description of physical systems, and allows proper scaling among different cases of practical interest.

We have extensively validated *FOCUS* with the *SRW* code. In particular, compared with standard multi-electron *SRW* simulations, *FOCUS* achieves a reduction in computation times by up to five orders of magnitude on a consumer laptop.

We have applied *FOCUS* to systematically characterize the transverse coherence properties of undulator radiation in typical third- and fourth-generation facilities. Results showed deviations from the well known van Cittert and Zernike theorem along the vertical direction when the electron beam emittance becomes comparable with, or smaller than, the natural photon beam emittance. While the observed discrepancies are small in current third-generation machines, they are however indicative of deviations that will become more evident in future fourth-generation light sources, especially close to the diffraction limit. In this case, the transverse coherence properties of the emitted radiation also largely vary across the radiation wavefront, and are affected by the peculiar features of the single-electron emission, in the presence of a finite, albeit small, emittance.

Finally, we remark that *FOCUS* is complementary to existing codes. It is aimed at fast evaluating the transverse coherence properties of synchrotron radiation from undulator sources as a function of the electron beam parameters, to support and help prepare more advanced and detailed numerical simulations with traditional codes like *SRW*.

*FOCUS* is publicly available from the website of the Instrumental Optics Laboratory of the Physics Department of Università degli Studi di Milano (https://instrumentaloptics.fisica.unimi.it/focus/). The source code is also freely available on the GitHub page of the corresponding author (https://github.com/MirkoSiano/FOCUS).

The code, specifically running on CUDA-capable NVIDIA graphics cards, has been tested on both Windows and Linux operating systems, but can be easily ported onto MacOS as well. In addition, pre-compiled executables are available on the *FOCUS* website, which makes manual compilation unnecessary. The executables allow the most common calculations to be performed, *e.g.* calculations of 1D profiles of the SDC. They will be continuously updated with new functionalities and cross-platform compatibility.

## Figures and Tables

**Table 1:** Main parameters of the NCD-SWEET beamline at ALBA; reduced quantities based on equations (2) and (11) are also reported

Quantity	Parameter	Value	Reduced parameter	Value
Electron beam energy	*E*	3 GeV		
Undulator strength parameter	*K*	1.56		
Undulator number of periods	*N*_*w*_	92		
Undulator period length	λ_*w*_	21.6 mm		
Harmonic number	*h*	7		
Observation distance	*z*	33 m	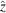	16.6
Horizontal beam size (r.m.s.)	σ_*x*_	130 µm	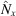	532
Horizontal beam divergence (r.m.s.)	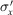	48 µrad	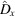	286
Vertical beam size (r.m.s.)	σ_*y*_	6 µm	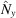	1.1
Vertical beam divergence (r.m.s.)	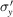	5 µrad	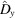	3.1
Energy spread (r.m.s.)	Δ_*E*_	1.05 × 10^−3^	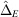	8.5

**Table 2:** Comparison between *FOCUS* and *SRW* simulation time as a function of the number of electrons in the beam *N*_e_

Code	*N*_e_ = 10	*N*_e_ = 10^2^	*N*_e_ = 10^3^	*N*_e_ = 10^4^	*N*_e_ = 10^5^	*N*_e_ = 10^6^
*FOCUS*	0.06 s	0.06 s	0.07 s	0.1 s	0.3–0.4 s	3–4 s
*SRW*	3 s	30 s	5 min	1 h	10 h	–

**Table 3:** Main parameters of the fourth-generation undulator source near the diffraction limit; reduced quantities based on equations (2) and (11) are also reported

Quantity	Parameter	Value	Reduced parameter	Value
Electron beam energy	*E*	6 GeV		
Undulator strength parameter	*K*	4.31		
Undulator number of periods	*N*_*w*_	72		
Undulator period length	λ_*w*_	69.4 mm		
Harmonic number	*h*	1		
Observation distance	*z*	30 m	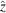	6
Horizontal beam size (r.m.s.)	σ_*x*_	4.47 µm	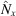	0.01
Horizontal beam divergence (r.m.s.)	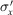	2.24 µrad	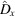	0.06
Vertical beam size (r.m.s.)	σ_*y*_	4.47 µm	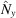	0.01
Vertical beam divergence (r.m.s.)	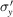	2.24 µrad	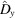	0.06
Energy spread (r.m.s.)	Δ_*E*_	1 · 10^−3^	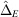	0.9
